# Blocking Jak/STAT signalling using tofacitinib inhibits angiogenesis in experimental arthritis

**DOI:** 10.1186/s13075-021-02587-8

**Published:** 2021-08-14

**Authors:** Paola Di Benedetto, Piero Ruscitti, Onorina Berardicurti, Noemi Panzera, Nicolò Grazia, Mauro Di Vito Nolfi, Barbara Di Francesco, Luca Navarini, Antonio Maurizi, Nadia Rucci, Anna Maria Teti, Francesca Zazzeroni, Giuliana Guggino, Francesco Ciccia, Vincenza Dolo, Edoardo Alesse, Paola Cipriani, Roberto Giacomelli

**Affiliations:** 1 Clinical Pathology Unit, Department of Biotechnological and Applied Clinical Sciences, University of L’Aquila, L’Aquila, Italy; 2 Division of Rheumatology, Department of Biotechnological and Applied Clinical Sciences, University of L’Aquila, L’Aquila, Italy; 3 Department of Biotechnological and Applied Clinical Sciences, University of L’Aquila, L’Aquila, Italy; 4 Unit of Rheumatology and Clinical Immunology, University of Rome “Campus Biomedico”, Rome, Italy; 5 Rheumatology Section, Department of Internal Medicine, University of Palermo, Palermo, Italy; 6 Rheumatology Section, Department of Clinical and Experimental Medicine, University of Campania “Luigi Vanvitelli”, Naples, Italy; 7 Clinical Pathology Unit, Department of Life, Health and Environmental Sciences, University of L’Aquila, L’Aquila, Italy

**Keywords:** Rheumatoid arthritis, Angiogenesis, Tofacitinib

## Abstract

**Objective:**

During rheumatoid arthritis (RA), the angiogenic processes, occurring with pannus-formation, may be a therapeutic target. JAK/STAT-pathway may play a role and the aim of this work was to investigate the inhibiting role of a JAK-inhibitor, tofacitinib, on the angiogenic mechanisms occurring during RA.

**Methods:**

After ethical approval, JAK-1, JAK-3, STAT-1, STAT-3 and VEGF expression was evaluated on RA-synovial-tissues. In vitro, endothelial cells (ECs), stimulated with 20 ng/ml of VEGF and/or 1 μM of tofacitinib, were assessed for tube formation, migration and proliferation, by Matrigel, Boyden chamber assay and ki67 gene-expression. In vivo, 32 mice received collagen (collagen-induced arthritis (CIA)) and 32 mice PBS (control). At day 19, CIA and controls mice were divided: 16 mice receiving vehicle and 16 mice receiving tofacitinib. At day 35, the arthritis score, the thickness of paw joints and the serum levels of VEGF and Ang-2 were evaluated.

**Results:**

The expression of JAK-1, JAK-3, STAT-1, STAT-3 and VEGF in synovial tissue of RA-patients were significantly higher than healthy controls. In vitro, tofacitinib inhibited the ECs ability to form vessels, to proliferate and to migrate. In vivo, administration of tofacitinib prevented the increase of the arthritis score, the paw thickness, the synovial vessels and VEGF and Ang-2 serum-accumulation, when compared to CIA without tofacitinib.

**Conclusions:**

We explored the anti-angiogenic role of tofacitinib, reporting its ability to inhibit in vitro the angiogenic mechanisms of ECs and in vivo the formation of new synovial vessels, occurring in CIA model. These findings suggest that the therapeutic effect of tofacitinib during RA may be also related to its anti-angiogenic activity.

**Supplementary Information:**

The online version contains supplementary material available at 10.1186/s13075-021-02587-8.

## Background

Rheumatoid arthritis (RA) is a chronic autoimmune disease, typically affecting the joints and associated with a significant morbidity [1]. A recent growing body of evidence has recently suggested a possible pathogenic loop between inflammation and angiogenesis [2, 3]. Indeed, it has been demonstrated that the infiltration of pro-inflammatory cells into the synovial tissues is associated with new vessels formation [2, 3]. The latter is a dynamic event based on two main processes, the vasculogenesis, defined as the differentiation of precursor cells (angioblasts) into endothelial cells (ECs) and the de novo formation of a primitive vascular network from progenitor ECs [2], and the angiogenesis, in which the new vessels develop from pre-existing blood vessels [2]. Mirroring what is observed in cancer, the rheumatoid pro-inflammatory process could enhance the synovial neo-vascularization, increasing immune cells infiltration, boosting itself and favouring the chronicity. Thus, as proposed in some anti-cancer therapeutic strategies [4, 5], the inhibition of angiogenesis could be a novel and attractive therapeutic strategy on RA with the possibility of reducing both synovitis and pannus formation. Intriguingly, in the last years, many works confirmed that some pro-angiogenic pathways involve Janus kinases/Signal Transducer and Activators of Transcription (JAK/STAT) which are also stimulated by the vascular endothelial growth factors (VEGF), a potent pro-angiogenic molecule [6–9]. The JAK family includes 4 members, JAK-1, JAK-2, JAK-3 and tyrosine kinase 2 (TYK2), which are variously combined with cytokines receptors to transmit cellular signalling [10]. Ligand/receptor binding promotes a conformational change of the receptor, which induces JAK activation, that further phosphorylates and activates the STAT proteins, acting as a transcription factor [11]. Thus, targeting JAK has been considered an effective therapy for RA, leading to the development of JAK inhibitors [11–13]. Recently, tofacitinib, a potent and selective JAK inhibitor, with a functional selectivity for signalling pathways mediated by JAK-1 heterodimers (JAK-1/3, JAK-1/2, JAK-1/Tyk-2), showed a good safety profile and efficacy on RA [14–17]. These effects have been correlated with an impairment of activation and proliferation of immune cells, via inhibition on pro-inflammatory cytokines, and the reduction of synovial phosphorylation of STAT-1 and STAT-3 [18].

Taking together these observations and considering the role of JAK/STAT in RA and new vessels formation, an unexplored therapeutic mechanism of targeting JAK/STAT may be the inhibition of neo-angiogenesis in rheumatoid pannus. Thus, in this work, we aimed to explore the angiogenesis in the synovial tissues of patients with RA and its inhibition by tofacitinib, by using human cells in a tri-dimensional scaffold. Furthermore, in an experimental model of arthritis, we also analysed the inhibition of angiogenesis by tofacitinib to assess its anti-angiogenic properties.

## Methods

### Patients and samples collection

The synovial tissues were obtained from 10 patients affected by RA, according to 2010 ACR/EULAR classification criteria and/or 1987 ACR criteria, and with an active disease (DAS28 > 3.2) [19, 20]. Full clinical data were reported in Table 1.

**Table 1:** Demographic and clinical characteristics of patients with RA and HC

	RA (10)	HC (10)
**Age at the time of biopsy (median years, range)**	56 (45–61)	58 (45–63)
**Gender (*n*, % of female)**	9 (90%)	8 (80%)
**Disease duration (median years, range)**	3 (1–5)	NA
**ACPA positivity (*n*, %)**	10 (100%)	NA
**RF status (*n*, %)**	10 (100%)	NA
**DAS28 (median, range)**	5.1 (4.8–5.9)	NA
**Low dosage of steroids (*n*, %)**	8 (80%)	NA
**Methotrexate (*n*, %)**	10 (100%)	NA

*RA* rheumatoid arthritis, *HC* healthy controls, *DAS28* Disease Activity Score on 28 joints, *ACPA* anti citrullinated peptide antibody, *RF* rheumatoid factor, *NA* not applicable

Normal synovial tissues were obtained from 10 matched healthy controls who underwent surgery due to a knee trauma. The local ethics committee approved the study protocol (ASL1, *Avezzano-Sulmona-L’Aquila, L’Aquila*, Italy, protocol number #36875) and it has been performed according to the Good Clinical Practice guidelines and the Declaration of Helsinki.

### Immunohistochemistry

Ten synovial tissues derived from RA patients and 10 synovial tissues derived from HC sections were examined for the expression of JAK-3 and STAT-1. Synovial sections (thickness 3 μm) were deparaffinised and treated with peroxidase-blocking reagent (DAKO, USA) to inactivate endogenous peroxidase and then with Protein block (DAKO, USA) to block non-specific binding. After blocking, sections were incubated with primary antibodies, including anti-JAK-3 and anti-STAT-1 (Abcam, UK). Visualisation of the primary antibodies was performed using DAB (diaminobenzidine) (DAKO, USA). Negative controls were obtained by omitting the primary antibody. Sections were examined and photographed under a light microscope (Olympus BX53). The optical density (OD) was measured by using NIH ImageJ version win64 freeware. Results were reported as the median (range) of OD/area.

### Immunofluorescence

Ten synovial tissues derived from RA patients and 10 synovial tissues derived from HC sections were examined for the expression of JAK-1, STAT-3, VEGF and vessel density. Synovial sections (thickness 3 μm) were deparaffinised and treated with protein block (DAKO, USA). After blocking, sections were incubated with primary antibodies, including anti-JAK-1 and anti-STAT-3 (Abcam, UK), anti-VEGF (Santa Cruz, USA) and anti vWF (Dako, Denmark). Visualisation of the primary antibodies was performed using Alexa Fluor 555- and 488-conjugated (Invitrogen, USA). Negative controls were obtained by omitting the primary antibody. The section was examined and photographed with an Olympus BX53 fluorescence microscope. The intensity of fluorescence was measured by using NIH ImageJ version 1.5 freeware. Vessel density was assessed by comparing the number of vessels VEGF+/vWF+ for field of synovial tissues.

### ECs coculture

Commercial healthy human microvascular ECs were cultured in completed MCDB131 medium (Sigma-Aldrich, USA) added with EGM-2 MV SingleQuotes (Lonza, BE). ECs were grown to confluence at 37 °C in 5% CO2.

### *In vitro* angiogenesis assay

Tube formation ability was evaluated using a Matrigel assay (BD, USA). Matrigel (9.2 mg/ml) was used at 1:1 dilution with a completed medium. ECs were seeded on Matrigel with VEGF (R&D, CDN) (20 ng/ml) and tofacitinib (1 μM). The concentration of tofacitinib followed what had already been published in similar experimental models [21–23]. After 4 h, the images were acquired using an Olympus BX53 microscope. The total tube length, the total branching length and the number of junctions of each experiment were measured, using NIH ImageJ. Results were expressed as median (range).

### Analysis of proliferation of ECs

ECs were treated with VEGF (20 ng/mL) and/or tofacitinib (1 μM), and the cellular expansion growth rate before and after tofacitinib treatment was evaluated by cell count in a Burker chamber and was expressed in terms of population-doubling (PD) using the formula: log n/log 2, where *n* is the P3 cell number of the confluent monolayer divided by the initial number of cells seeded at P2. Furthermore, the Ki67 gene expression, which is strictly associated with cell proliferation [24] was evaluated by immunofluorescence.

### Cells immunofluorescence

Cells (5 × 10^3^) were grown in 8 wells culture slides (BD, USA) in a completed medium for 24 h, and then they were starved for 24 h in 0.5% foetal bovine serum (FBS) (Lonza, BE) medium. Successively, the cells were stimulated with 20 ng/mL of VEGF and/or tofacitinib (1 μM) for 24 h. The untreated (UT) cells were maintained at the same conditions of the treated cells. For the staining, the cells were fixed with 4% paraformaldehyde (EMS, PA), incubated 20 min with protein block (DAKO, USA) and successively with an anti-human ki67 antibody (Invitrogen, USA). The visualisation of the primary antibodies was performed using Alexa Fluor 555-conjugated (Invitrogen, USA). After counterstained using 4′, 6-diamidino-2-phenylindole (DAPI), images were acquired using an Olympus BX53 fluorescence microscope. The number of ki67+ cells was counted and results were reported as the median (range) of a number of positive cells per microscopic field.

### Chemoinvasion assay

ECs chemoinvasion was evaluated by 48-well modified Boyden chamber. We used filters (8 mm) coated with Matrigel. For the evaluation of the basal motility, 199 medium supplemented with 0.5% FBS was used in the lower chamber. After treatment with the VEGF (20 ng/mL) and/or tofacitinib (1 μM), the cells were added to the upper chamber at a density of 8 × 10^3^ cells per well, suspended in 199 media containing 2% foetal bovine serum. After 6 h of incubation at 37 °C, the non-migrated cells on the upper surface of the filter were removed by scraping. The cells that migrated to the lower side of the filter were stained with Diff-Quick stain and counted, using an Olympus BX53 microscope. The assays were run in triplicate. Results were reported as median (range) of a number of cells migrated per microscopic field.

### Murine experimental model

Sixty-four specific pathogen-free, female DBA/1 J mice, 6 weeks old, mean body weights 17.5 ± 1.1 g, were purchased from the Jackson Laboratory USA and fed ad libitum. Procedures involving animals and their care were conducted in conformity with National and International Laws and Policies (European Economic Community Council Directive 86/609, OJ L 358, 1, December 12, 1987; Italian Legislative Decree 4.03.2014, n.26, *Gazzetta Ufficiale della Repubblica Italiana* no. 61, March 4, 2014) and approved by Italian Ministry of Health (179/2018-PR). The sample size of mice was elaborated considering two main features: (i) the lack of previous evidence about the effects of tofacitinib on angiogenesis, comparing collagen-induced arthritis (CIA) mice treated with tofacitinib vs CIA mice treated without tofacitinib and comparing control mice treated with tofacitinib vs control mice treated without tofacitinib; and (ii) the necessity to minimise the sample size for ethical reasons, establishing the power 1-β = 90% and the statistical significance *α* = 0.05%. The effect size was chosen according to Cohen’s criteria [25], considering Δ = δ/σ≥0.8 and Δ =1. The calculation provided the following results: group of CIA mice: *n*_tofacitinib_ = 16; *n*_no-tofacitinib_ = 16; group of control mice: *n*_tofacitinib_ = 16; *n*_no-tofacitinib_ = 16; 1-β = 0.90; *α* = 0.05. The randomisation of the mice in 4 different groups (*N* = 16) was performed, at day 0, before the start with protocol, using a computer-based random order generator. Considering all these features, the sample size resulted to be *n* = 32 for the CIA mice and *n* = 32 for the control mice. Before starting with the procedure, animals were randomised into 4 different groups (*N* = 16).

### Collagen-induced arthritis (CIA) model

For the CIA model, 100 μg of bovine type II collagen (Chondrex, USA), dissolved in 0.1 M acetic acid (Sigma-Aldrich, USA), was emulsified with an equal volume of Freund’s complete adjuvant (Chondrex, USA) and administered intra-dermally at the base of the tail into DBA/1 J mice (CIA mice, *n* = 32). Following the same procedure, a control group received saline solution in place of type II collagen (control mice, *n* = 32). At day 18, CIA mice received a booster emulsion prepared with type II collagen and Freund’s incomplete adjuvant, control mice received a saline solution, and all the solutions were administered intra-dermally, near the primary injection. At day 19, controls and CIA mice were divided into two subgroups: one receiving vehicle (*n* = 16) and one receiving 30 mg/kg/day of tofacitinib (*n* = 16). The administration of the vehicle and the tofacitinib was performed via oral gavage. Thirty-five days after the first collagen administration, the mice were sacrificed and the blood collected. At day 35, before the sacrifice, the arthritis score was assessed and was monitored by one blinded operator, measuring the thickness of paw joints, using a dial-type calibre and reported as the median (range) of mm of every group of treatment. A schematic representation of in vivo experiments was reported in supplementary material 1.

### Arthritis score assessment

Arthritis score was evaluated by one blinded operator, as 5 scales as previously reported [26]: (i) 0: no evidence of erythema and swelling; (ii) 1: erythema and mild swelling confined to the tarsals or ankle joint; (iii) 2: erythema and mild swelling extending from the ankle to the tarsals; (iv) 3: erythema and moderate swelling extending from the ankle to metatarsal joints; and (v) 4: erythema and severe swelling encompass the ankle, foot and digits or ankylosis of the limb. Arthritis was scored using a scale of 0–4 for each paw.

### Histological analysis in mice of synovial vascular density

Whole knee joints from mice were dissected and fixed in 10% buffered formalin (Bioptical, Italy) for 1 day, decalcified in Osteodec (Bioptical, Italy) for 1 day, and successively dehydrated and embedded in paraffin blocks. For haematoxylin and eosin (H&E) staining, the slides were dewaxed, rehydrated and stained with H&E. In order to measure vessel density in synovial membrane tissues of joints, the polyclonal antibody anti-rabbit-von Willebran factor (vWF) (DAKO, USA) was used for micro-vessels staining on 5-mm-thick paraffin-embedded sections of knee joints. The immunohistochemistry was performed as above reported. Vessel density was assessed by one blinded operator, counting the number of vessels vWF+ for the field of synovial membrane tissue.

### ELISA assay on mouse sera

The concentration of VEGF and Ang-2, released in the serum of CIA mice and control mice, was determined by ELISA (for VEGF, CDN, USA, for Ang-2, MyBiosource, USA), according to the manufacturer’s protocol.

### Statistical analysis

GraphPad Prism 5.0 software was used for all statistical analyses. Due to the non-parametric distribution, results were expressed as median (range) and Mann-Whitney *U* test was used as appropriate for analyses. Statistical significance was expressed by a *p* value < 0.05.

## Results

### JAK-1, JAK-3, STAT-1, STAT-3 and VEGF expression in synovial tissues of patients with RA

**Fig. 1:**
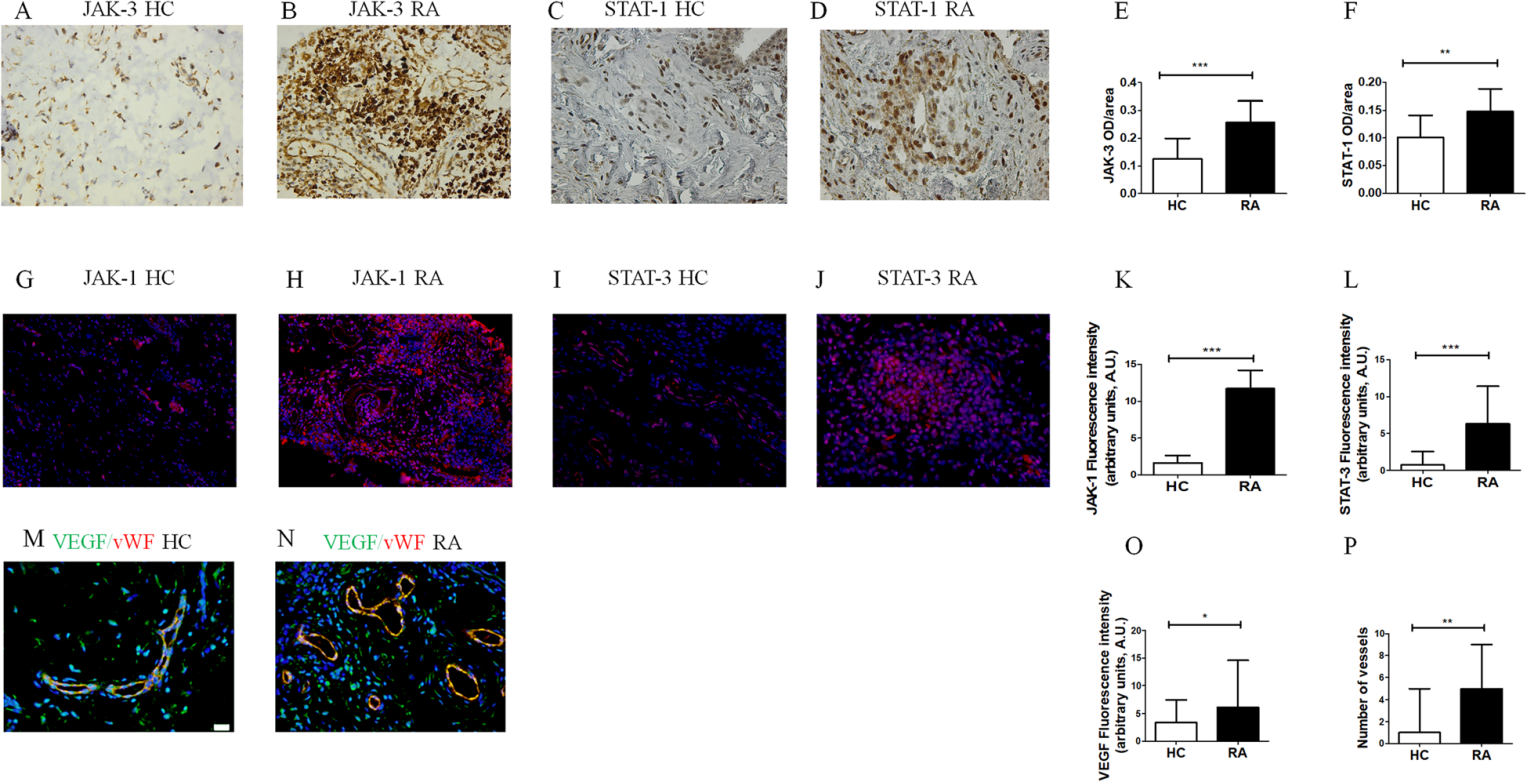
JAK-1/3 and STAT-1/3 expression in synovial tissue of patients with RA. **A**–**D** JAK-3 (**A**) and STAT-1 (**C**) staining of HC synovial tissue; JAK-3 (**B**) and STAT-1 (**D**) staining of RA synovial tissue. **E**, **F** The optical density (OD)/area of JAK-3 (**E**) and STAT-1 (**F**) were significantly higher in RA synovial tissue when compared with HC synovial tissue. The histogram showed median and range of OD/area for each synovial tissue (**p = 0.006; ***p = 0.0003). **G**–**J** JAK-1 (**G**) and STAT-3 (**I**) staining of HC synovial tissue; JAK-1 (**H**) and STAT-3 (**J**) staining of RA synovial tissue. **K**, **L** The fluorescence intensity of JAK-1 (**K**) and STAT-3 (**L**) were significantly higher in RA synovial tissue, when compared with HC synovial tissue. The histograms showed median and range of fluorescence intensity of each synovial tissues (**p ≤ 0.0002). **M**, **N** VEGF (green) and vWF (red) staining of HC (**M**) synovial tissue and RA (**N**) synovial tissue. **O**, **P** The fluorescence intensity of VEGF (**O**) and the number of vessel expressing vWF (**P**) were significantly higher in RA synovial tissue, when compared with HC synovial tissue. The histograms showed median and range of each synovial tissue (*p = 0.02; **p = 0.001). Original magnification ×20

As shown in Fig. 1, JAK-1 and JAK-3 (panels A, B, G, H) and STAT-1 and STAT-3 (panels C, D, I, J) were expressed in vascular cells and inflammatory cells. The values of OD/area of JAK-3 and STAT-1 were significantly increased in RA synovial tissues, when compared to HCs (JAK-3, *p* = 0.003; STAT-1, *p* = 0.007), as observed in Fig. 1E and F. Furthermore, the results showed that in RA, the intensity of fluorescence of JAK-1 and STAT-3 was significantly increased when compared to HCs (JAK-1, *p* < 0.0001; STAT-3, *p* = 0.0002). As shown in Fig. 1M and N, VEGF was expressed by vascular cells and fibroblasts of synovial tissues. In RA, the intensity of fluorescence of VEGF was significantly increased when compared to HCs (*p* = 0.02). Furthermore, in RA, the number of cells expressing both vWF and VEGF was significantly higher when compared to HCs (*p* = 0.001).

### The inhibition of in vitro angiogenesis by tofacitinib

**Fig. 2:**
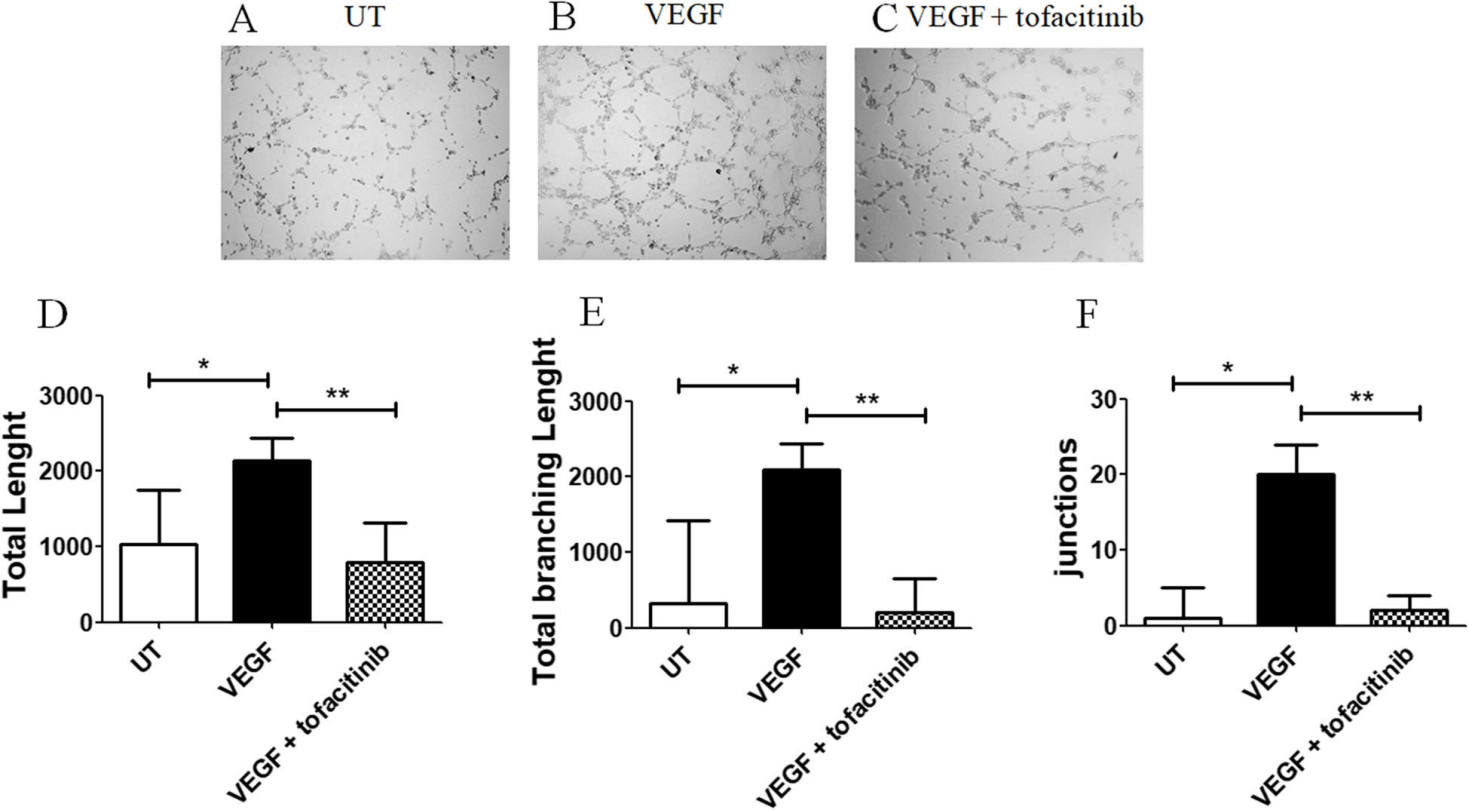
Effect of Tofacitinib on tube formation of HC-ECs. **A**–**C** Representative image of Matrigel angiogenesis assays of untreated (UT) HC-ECs (**A**), HC-ECs treated with 20 ng/ml of VEGF (**B**) and HC-ECs treated with 20 ng/ml of VEGF and 1 μM of tofacitinib (**C**). **D**–**F** Quantification of total length (**D**), total branching length (**E**) and junctions (**F**) of the tubes obtained in the in matrigel assay. In HC-ECs, the stimulation with VEGF induced a significant increase in the total tube length, the total branching length and the number of junctions, when compared to UT HC-ECs. In HC-ECs treated with both tofacitinib and VEGF, the total tube length, the total branching length and the number of junctions were significantly decreased, when compared with HC-ECs treated with VEGF. The histograms showed the median and the range of total tube length, total branching length and number of junctions in matrigel assay (*p ≤ 0.03; **p = 0.007)

In our in vitro Matrigel assay, the stimulation of HC-ECs with 20 ng/ml of VEGF (Fig. 2B), induced the formation of well organised tube-like structures. In fact, in this condition, the total tube length (Fig. 2D), the total branching length (Fig. 2E) and the number of junctions (Fig. 2F) were significantly increased when compared to UT HC-ECs [total tube length in UT HC-ECs 1029 (489–1755) vs total tube length in VEGF HC-ECs 2136 (1405–2437), *p* = 0.03; total branching length in UT HC-ECs 320 (0–1421) vs total branching length in VEGF HC-ECs 2100 (1101–2437), *p* = 0.03; the number of junctions in UT HC-ECs 1 (0–5) vs number of junctions in VEGF HC-ECs 20 (6–24), *p* = 0.01]. Following the stimulation with both tofacitinib and VEGF, HC-ECs decreased their ability to form well-organised vessels. In fact, the total tube length, the total branching length and the number of junctions were significantly decreased when compared to VEGF HC-ECs [total tube length in tofacitinib + VEGF HC-ECs 796 (711–1308) vs total tube length in VEGF HC-ECs 2136 (1405–2437), *p* = 0.007; total branching length in tofacitinib + VEGF HC-ECs 208 (115–648) vs total branching length in VEGF HC-ECs 2100 (1101–2437), *p* = 0.007; number of junctions in tofacitinib+VEGF HC-ECs 2 (1–4) vs number of junctions in VEGF HC-ECs 20 (6–24), *p* = 0.007].

### The inhibition of chemoinvasion and proliferation of HC-ECs by tofacitinib

**Fig. 3:**
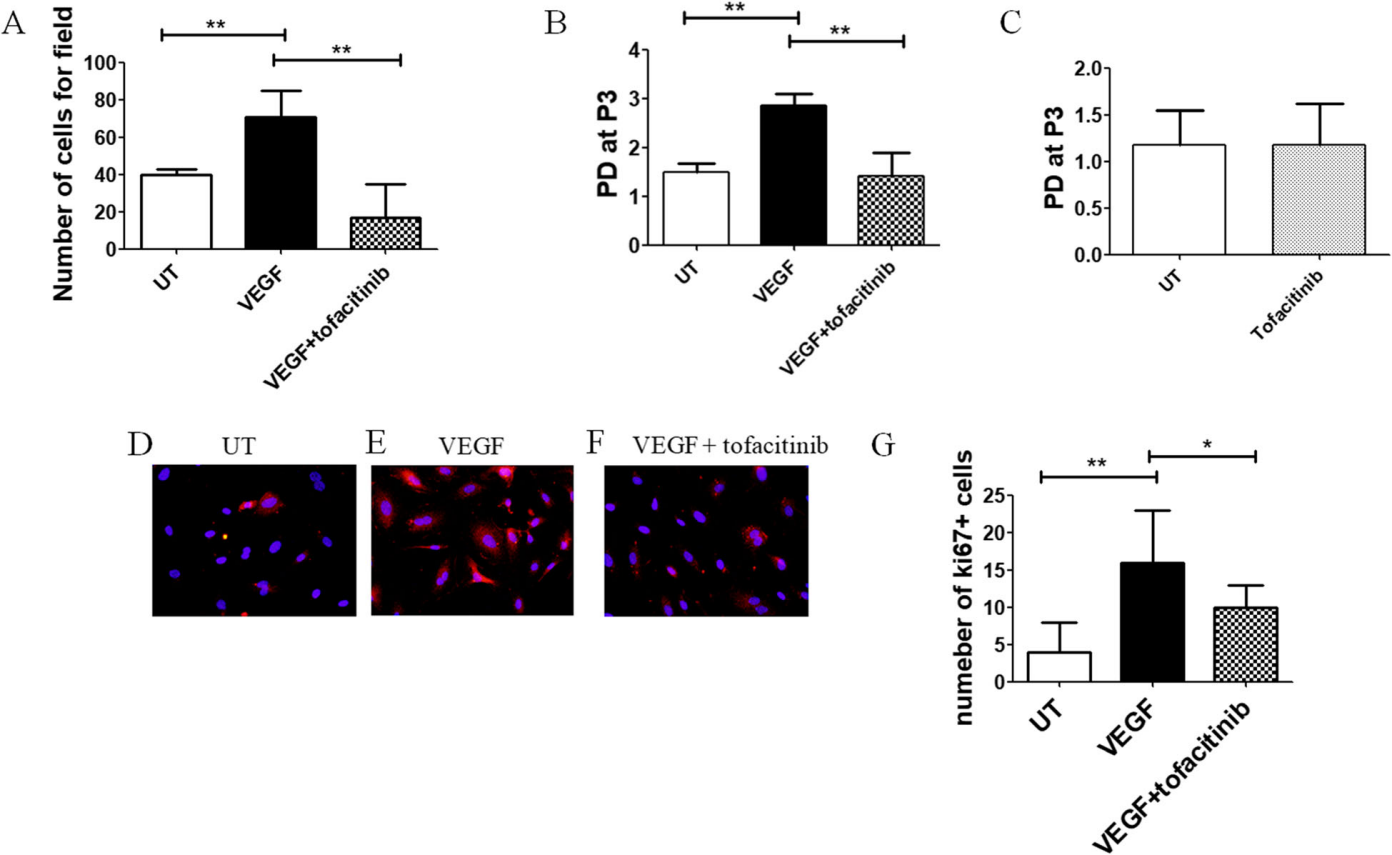
Effect of tofacitinib on chemoinvasion and proliferation of HC-ECs. (**A**) The histogram showed the median and the range of migrated HC-ECs. VEGF (20 ng/ml) significantly induced an increase of number of migrated HC-ECs, when compared untreated (UT) HC-ECs and the treatment with 1 μM of tofacitinib significantly decrease the effects of VEGF (**p = 0.007). (**B**)The histogram showed the median and the range of cumulative population-doubling (PD) levels. VEGF (20 ng/ml) significantly induced an increase of PD in HC-ECs, when compared untreated (UT) HC-ECs and the treatment with 1 μM of tofacitinib significantly decreased the effects of VEGF (**p = 0.007). (**C**) The histogram showed the median and the range of cumulative population-doubling (PD) levels. The PD of ECs treated with Tofacitinib 1 μM was comparable to PD of UT cells. **D**–**F** Representative image of immunofluorescence of ki67 expression (red) in HC-ECs cultured in untreated (UT) condition (**D**), treated with VEGF (**E**) and treated with VEGF+tofacitinib (**F**). Negative controls were obtained by omitting the primary antibody. Original magnification ×20. **G** The histogram showed the median and the range of the number of HC-ECs expressing ki67. VEGF significantly induced an increase of the number of HC-ECs ki67+, when compared to untreated (UT) HC-ECs and the treatment with tofacitinib significantly decreased the effects of VEGF (*p = 0.03; **p = 0.007)

We found that HC-ECs invasion was significantly induced by VEGF (20 ng/ml), when compared with UT HC-EC [number of migrated VEGF HC-ECs 70.7 (53.0–84.7) vs number of migrated UT HC-ECs 40.0 (24.3–42.7), *p* = 0.007]. The effect of VEGF was reverted by the stimulation of the cells with tofacitinib [(number of migrated tofacitinib + VEGF HC-ECs 16.7 (11.7–35.0) vs number of migrated VEGF HC-ECs 70.7 (53.0–84.7), *p* = 0.007] (Fig. 3A). Furthermore, to assess the ability of tofacitinib to prevent the proliferation of HC-EC treated with VEGF, we evaluated the PD, as markers of the replication rate. Figure 3B showed that the stimulation with VEGF (20 ng/ml) induced a significant increase of PD than UT HC-ECs [PD of VEGF HC-ECs 2.8 (2.7–3.0) vs PD of UT HC-ECs 1.4 (1.0–1.6), *p* = 0.007]. The effect of VEGF was reverted by the stimulation of the cells with tofacitinib [PD of tofacitinib + VEGF HC-ECs 1.4 (0.7–1.9) vs PD of VEGF HC-ECs 2.8 (2.7–3.0), *p* = 0.007]. Additionally, when ECs were stimulated with tofacitinib alone, the PD was comparable to UT cells, thus excluding its toxic effect [PD of tofacitinib HC-ECs 1.2 (1.0–1.6) vs PD of UT HC-ECs 1.2 (1.1–1.5), *p* = 0.92] (Fig. 3C). The proliferation of HC-ECs was also evaluated by immune-fluorescence, assessing the ki67 expression, a molecule associated with active proliferation. Figure 3D–G showed that the treatment with VEGF induced a significant increase of the number of ki67+ HC-ECs when compared to UT cells [number of ki67+ cells in VEGF HC-ECs 16 (10–23) vs number of ki67+ cells in UT HC-ECs 4 (1–8), *p* = 0.007]. The effect of VEGF was reverted by tofacitinib [number of ki67+ cells in VEGF + tofacitinib HC-ECs 10 (3–13) vs number of ki67+ cells in VEGF HC-ECs 16 (10–23), *p* = 0.03].

### Tofacitinib inhibits arthritis and angiogenesis in CIA mice

**Fig. 4:**
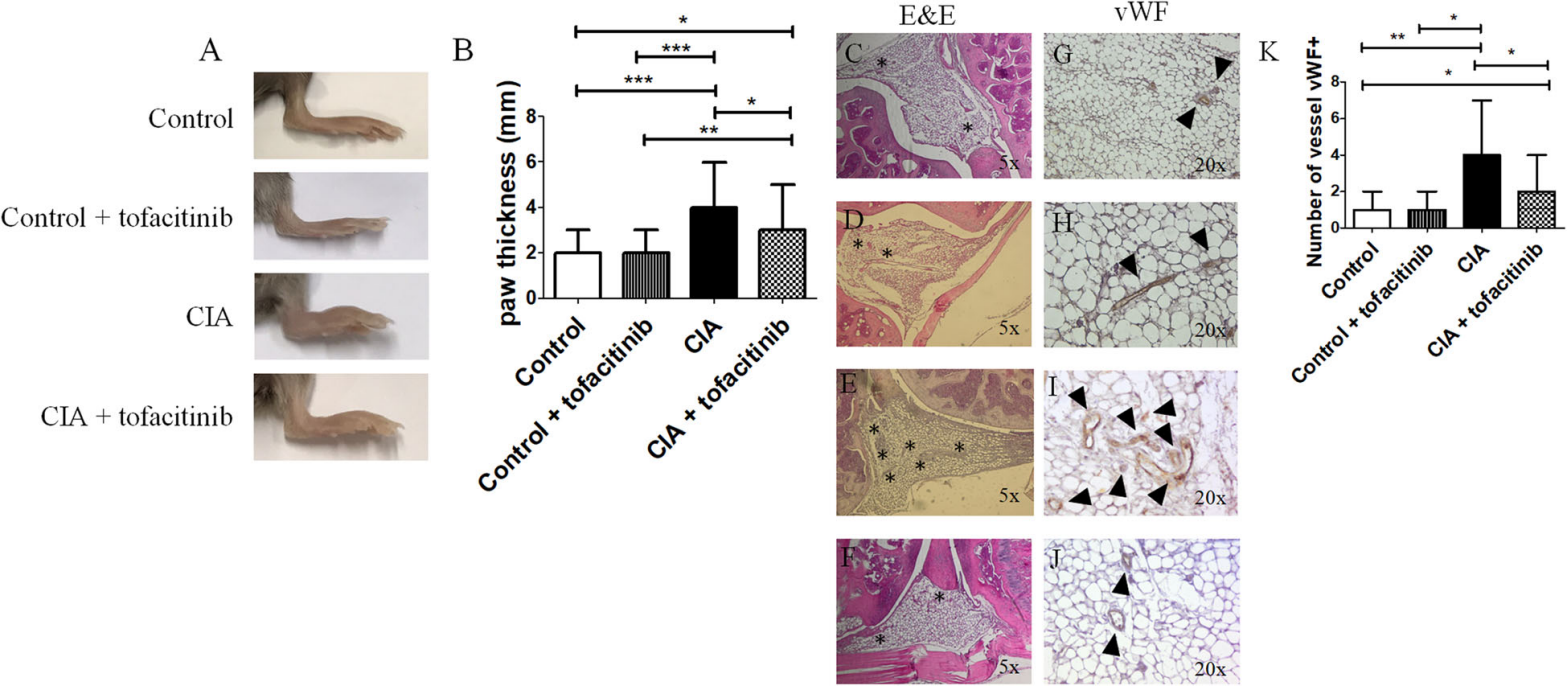
Tofacitinib effects on the in vivo model of experimental arthritis. (**A**) Representative image of the paw of control mice and CIA mice, treated with and without tofacitinib. (**B**) The histogram showed the median and the range of the paw thickness (mm) of the mice. The collagen induced a significant increase of paw thickness when compared to the control group, and 30 mg/kg/day of tofacitinib prevented the increase of paw thickness (*p ≤ 0.03; **p = 0.007; ***p = 0.0001). **C**–**F** Representative image of haematoxylin and eosin (H&E) staining of synovial tissues from knee joints derived from control mice (**C**), control mice treated with tofacitinib (**D**), CIA mice (**E**) and CIA mice treated with tofacitin (**F**). The micro-vessels are highlighted by *. Original magnification 5×. **G**–**J** Non-consecutive representative image of vWF staining of synovial tissues from knee joints derived from control mice (**G**), control mice treated with tofacitinib (**H**), CIA mice (**I**) and CIA mice treated with tofacitin (**J**). The pictures showed the staining of vWF, used for micro-vessels staining (arrows). Original magnification 20×. (**K**) Quantification of the number of vessels expressing vWF+. The treatment of CIA mice with tofacitinib significantly reduced the vessel density in synovial tissues of joints, when compared to CIA that did not receive tofacitinib. The histograms showed the median and range of each synovial tissue (*p < 0.04; **p = 0.009)

We assessed the effect of tofacitinib in CIA DBA1/J mice. After 35 days from the first collagen administration, the arthritis score was assessed to evaluate that the arthritis disease process had been established. In CIA mice, the arthritis score was significantly higher when compared to control mice [arthritis score in CIA mice 7 (3–9) vs arthritis score in control mice 0 (0–1), *p* < 0.0001] (Supplementary material 2). Oral administration of tofacitinib 30 mg/kg started from day 19 and continued once a day until day 34. In the group of CIA mice treated with tofacitinib, the arthritis score was significantly lower when compared to CIA mice treated with vehicle [arthritis score in CIA + tofacitinib mice 4 (2–8) vs arthritis score in CIA mice 7 (3–9), *p* = 0.001] (Supplementary material 2). Furthermore, we evaluated the paw thickness of the mice; Fig. 4A and B reported that in the group of mice receiving the collagen (CIA) paw thickness was significantly increased when compared with the mice which did not receive the collagen administration (control) [paw thickness of CIA mice 4 mm (2–6) vs paw thickness of control mice 2 mm (1.5–3), *p* = 0.0001]. Tofacitinib significantly prevented the increase of paw thickness induced by the collagen administration [paw thickness of CIA mice+ tofacitinib 3 mm (2–5) vs paw thickness of CIA mice 4 mm (2–6), *p* = 0.02] (Fig. 4B). The immunohistochemical analysis (Fig. 4C, D) showed that oral administration of tofacitinib in CIA mice significantly reduced the vessel density in synovial tissues of joints, when compared to CIA mice not treated with tofacitinib [number of vessels vWF+ in CIA mice + tofacitinib 2 (1–4) vs number of vessels vWF+ in CIA mice 4 (2–7), *p* = 0.03].

### Serum levels of VEGF and Ang-2 in CIA mice

**Fig. 5:**
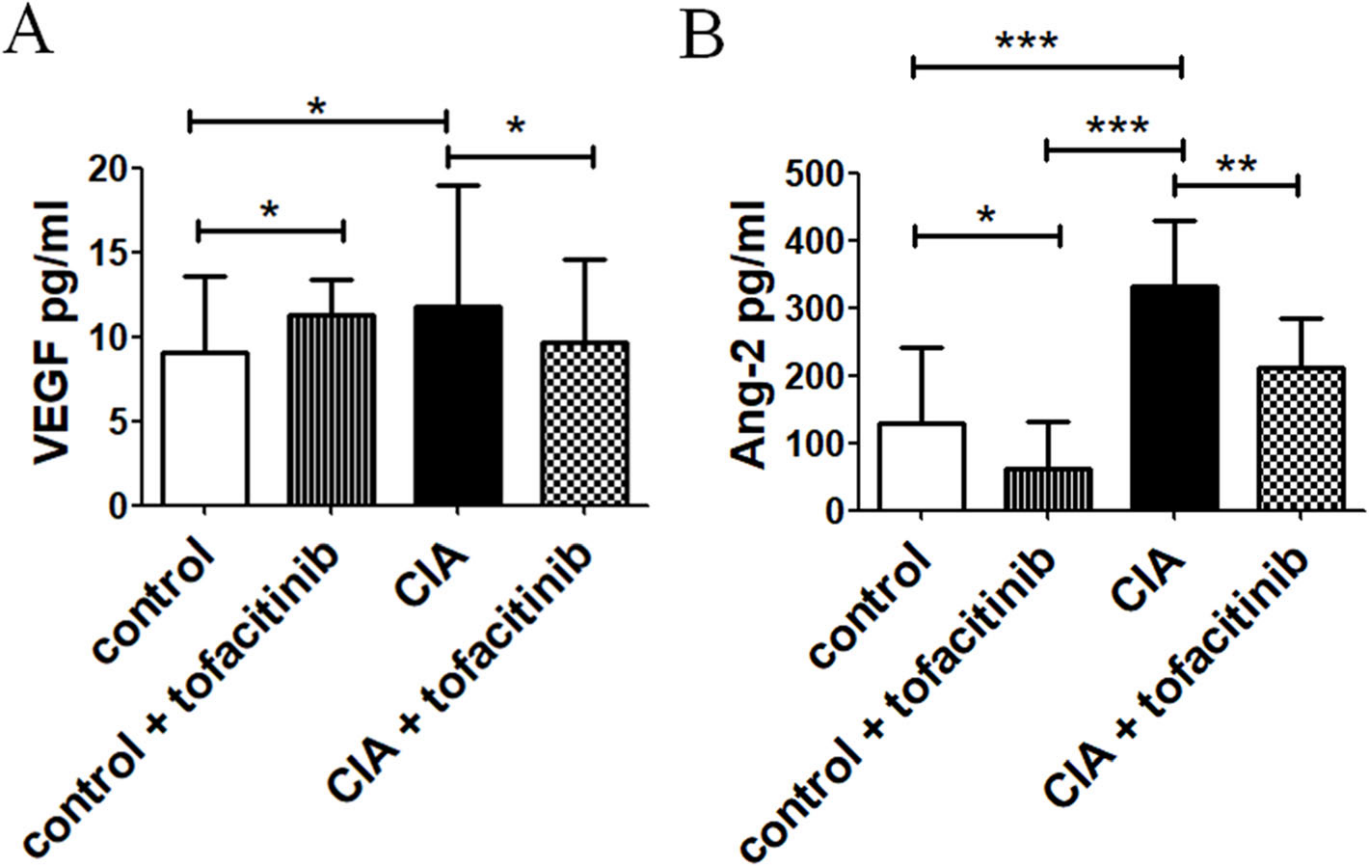
Tofacitinib effects on serum levels of VEGF and Ang-2 in vivo model of experimental arthritis. **A** ELISA assay of VEGF. The serum levels of VEGF in CIA mice were significantly increased, when compared to control mice. Tofacitinib prevented the increase of serum levels of VEGF, when compared to CIA mice untreated with tofacitinib (*p ≤ 0.04). **B** The serum ELISA assay of Ang-2. The serum levels of Ang-2 in CIA mice were significantly increased, when compared to control mice. Tofacitinib prevented the increase of serum levels of Ang-2, when compared to CIA mice untreated with tofacitinib (*p = 0.01; **p = 0.002; ***p ≤ 0.0001)

Serum levels of VEGF and Ang-2 were higher in CIA mice, than in control mice [VEGF pg/ml in CIA mice 11.7 (9.2–18.9) vs VEGF pg/ml in control mice 9.0 (6.6–13.5), *p* = 0.01] (Fig. 5A). The administration of tofacitinib reduced the VEGF accumulation in CIA mice [VEGF pg/ml in CIA mice + tofacitinib 9.6 (5.4–14.6) vs VEGF pg/ml in CIA mice 11.7 (9.2–18.9), *p* = 0.03]. Furthermore, in CIA mice, the serum levels of Ang-2 were significantly increased when compared to control mice, and the stimulation with tofacitinib prevented this increase [Ang-2 pg/ml in CIA mice 332.5 (237.6–430.2) vs Ang-2 pg/ml in control mice 128.9 (40.3–241.2), *p* < 0.0001; Ang-2 pg/ml in CIA mice + tofacitinib 211.8 (136.7–283.1) vs Ang-2 pg/ml in CIA mice 332.5 (237.6–430.2), *p* = 0.002] (Fig. 5B).

## Discussion

In this work, we explored the anti-angiogenic role of tofacitinib, reporting that this drug inhibited in vitro the angiogenesis and the proliferation of ECs and prevented in vivo the formation of new synovial vessels in a murine model of arthritis. These findings suggest that the therapeutic effect of tofacitinib during RA may be also related to its anti-angiogenic activity.

In RA synovial tissues, the OD of JAK-3 and STAT-1 and the intensity of fluorescence of JAK-1 and STAT-3 were significantly increased when compared with HCs, mirroring previous works on JAK/STAT involvement on this disease [18, 21, 22, 27]. Furthermore, the VEGF, one of the main pro-angiogenic growth factors [28, 29], was significantly increased in RA than in HCs. Interestingly, in these tissues, vessels vWF+VEGF+ were more represented than HCs. These results could suggest that the new vessel formation in RA is increased paralleling with an increased JAK/STAT expression.

Furthermore, we assessed the effects of tofacitinib on specific steps of angiogenesis; finding that this drug inhibited tube formation, migration and proliferation of HC-ECs, reducing the pro-angiogenic effects of VEGF. In fact, tofacitinib impaired total tube lengths, total branching lengths and the number of junctions, thus reducing the formation of well-organised vessels. Similarly, tofacitinib reduced the VEGF-induced proliferation and migration of ECs, which precede the structural reorganisation of ECs into a three-dimensionally tubular structure. Based on our results, it is possible to postulate that tofacitinib influenced all the phases of vascular network formation. Despite the signalling of VEGFR2 is reported to be mediated by JAK-2 [7, 8, 30] and STAT-3 proteins [31, 32], we observed an interference of tofacitinib on the VEGF functions. In this context, tofacitinib is a selective JAK-1 heterodimers inhibitor (JAK-1/3, JAK-1/2, JAK-1/Tyk-2), affecting heterodimers comprising also the JAK-2 [33, 34], which is implicated in VEGFR2 signalling [7, 8, 30]. In addition, tofacitinib efficiently blocked granulocyte-macrophage colony-stimulating factor (GM-CSF)-induced JAK-2 phosphorylation in human neutrophils [35], confirming recent evidence about possible pan-JAK inhibition by this drug [13, 36–38]. Furthermore, the evidence that tofacitinib could prevent the proliferation of ECs. This finding paralleled with previous results, showing that this drug could induce a cell cycle arrest and inhibition on cell growth in NK92 and STAT3-mutant cells [39, 40].

To translate these in vitro results in an in vivo experimental model of RA, we used a CIA model of arthritis [26]. The results showed that the administration of tofacitinib to CIA mice significantly prevented the development of arthritis, confirming what was previously reported [16, 41, 42]. To our data, the occurrence of arthritis matched with the development of an aberrant angiogenesis and hyperproduction of VEGF and Ang-2 levels, which, in the presence of abundant VEGF, is able to promote vascular sprouts by blocking the maturation and stabilisation processes of new vessels [43–47]. In fact, the synovial vessels density in the arthritis joint of CIA mice was significantly increased when compared to control mice. Of interest, our results showed that tofacitinib reduced the vessel density in arthritis joint of CIA mice and the sera VEGF and Ang-2 levels, confirming our in vitro results about its anti-angiogenic properties. These data paralleled previous work, reporting an anti-angiogenic role of tofacitinib in an experimental model of giant cell arteritis, in which this drug effectively suppressed the microangiogenic growth of capillary networks and intimal hyperplasia [48]. Additionally, our in vivo results suggest that the clinical efficacy of tofacitinib in RA could be also related to the inhibition of neo-angiogenesis in the rheumatoid pannus. In fact, aberrant angio-architecture and impaired vascular functionality are essential for pannus formation and damage progression since the new blood vessels may provide essential nutrients and oxygen to pro-inflammatory and proliferative cells [49–52]. In fact, in previous work, it has been reported that JAK-inhibitor may inhibit the hypoxia-inducible factor-1α (HIF-1α) signalling angiogenic mechanisms [53, 54]. On the other hand, it is also possible that a better control of inflammation may indirectly decrease the angiogenesis in the synovial tissue. However, without excluding the anti-inflammatory property of tofacitinib, we clearly documented an anti-angiogenic ability of this drug, in our in vitro experiments where a decreased of tubes was observed. In addition, the good clinical results of JAK inhibition during RA may also be explained by a synergistic effect between the anti-angiogenic and anti-inflammatory activities.

We are aware that in this study, there are some limitations, such as the higher tofacitinib dosage used during in vivo animal experiments (30 mg/kg/day) when compared with the dosages in clinical practice (the maximum dosage approved for human is 10 mg/day which, assuming the average patient weighs 70 kg, corresponds to 0.15 mg/kg/day) [55]. In this study, the dosage of tofacitinib for the treated mice was chosen, according to a previous published paper [43], in which 30 mg/kg/day have been shown as the optimal dosage to significantly prevent the development of a severe disease in CIA mice. On this basis, the high dose administered in mice may potentially causes off-target effects, in humans. Thus, further studies are needed to evaluate the possible anti-angiogenic effect of tofacitinib, by using lower dosages of such drug, to evaluate the magnitude of these anti-angiogenic effects and their possible translational application in human therapy. Additionally, another limitation is related to the relatively low number of observations which could limit the external validity of our findings. However, our sample estimation was based on the lack of previous evidence about the effects of tofacitinib on angiogenesis and was based on the necessity to minimise the sample size for ethical reasons. Considering the non-normally distribution of the results, we expressed our results as median and range; for summary statistics of continuous data with an asymmetrical distribution, the median has been found to reflect the distribution more accurately than the mean [56]. Furthermore, we used a non-parametric *T* test to assess our results since data substantially deviated from normality. In our study, our results were statistically significant despite the sample sizes was too small to satisfy the condition of the central limit theorem because of the relatively low number of observations related to the specific sample size calculation. Taking together these observations and the lack of previous evidence about the effects of tofacitinib on angiogenesis, our work may be considered a hypothesis-generating study needing to be subsequently confirmed. In fact, although murine models of arthritis may have a high relevance to study the pathogenic steps involved in arthritides development and progression, none of the experimental models used may entirely recapitulate the clinical pathology of RA [57].

## Conclusions

In conclusion, during the last decade, the biological analogies between cancers and synovial pannus, lead to an increasing interest for angiogenesis as a possible therapeutic target in RA. The present study could show the anti-angiogenic properties of tofacitinib, describing a still not fully explored mechanism of action for this molecule which surely may play an important role in controlling the clinical evolution of RA and supporting the hypothesis that angiogenesis and inflammation should possibly be targeted together. However, considering the limitations of our work, further studies are needed to fully clarify this issue.

## Supplementary Information

**Additional file 1: Supplementary material 1.** Mice treatments. The first day (day 0) of the procedure, 64 DBA/1 J mice were divided in 2 groups. One control group (*n*=32) receiving saline solution and one CIA group (*n*=32) receiving 100 μg of bovine type II collagen, emulsified with an equal volume of Freund’s complete adjuvant. After 18 days, the control group received saline solution and CIA mice received type II collagen and Freund’s incomplete adjuvant. At the day 19, controls and CIA mice were divided into 2 subgroups: one receiving vehicle (*n*=16) and one receiving 30 mg/kg/day of tofacitinib (*n*=16). After 35 days the first collagen administration, the mice were sacrificed and the blood collected.

**Additional file 2: Supplementary material 2.** Arthritis score evaluation. The histogram showed the median and the range of the arthritis score evaluated the day 35. The collagen induced a significant increase of arthritis score when compared to control group, and 30 mg/Kg/day of tofacitinib prevented the increase of arthritis score (**=*p*=0.001; ***= *p*< 0.0001).

## Data Availability

Relevant files of this work will be shared at reasonable request.

## Abbreviations

CIA: Collagen-induced arthritis
ECs: Endothelial cells
GM-CSF: Granulocyte-macrophage colony-stimulating factor
HIF-1α: Hypoxia-inducible factor-1α
JAK/STAT: Janus kinases/Signal Transducer and Activators of Transcription
RA: Rheumatoid arthritis
TYK2: Tyrosine kinase 2
VEGF: Vascular endothelial growth factors
vWF: von Willebran factor
