# Supplementary figures and images for: Blocking Jak/STAT signalling using tofacitinib inhibits angiogenesis in experimental arthritis
Source: Arthritis Res Ther. 2021 Aug 14;23:213. doi: 10.1186/s13075-021-02587-8 (PMC8364029; doi:10.1186/s13075-021-02587-8)

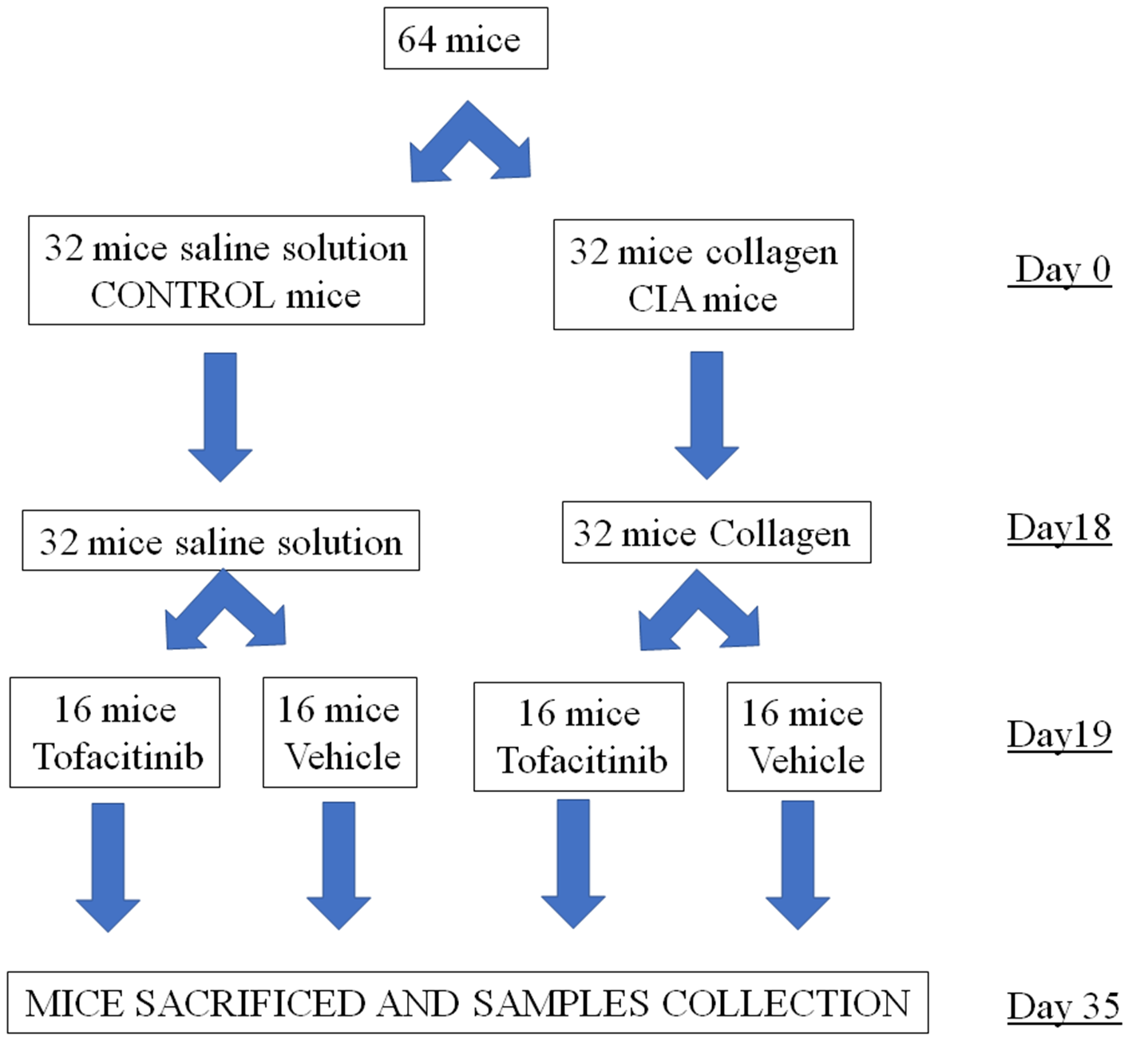

Supplement: Supplementary file 1 — Additional file 1: Supplementary material 1. Mice treatments. The first day (day 0) of the procedure, 64 DBA/1 J mice were divided in 2 groups. One control group (n=32) receiving saline solution and one CIA group (n=32) receiving 100 μg of bovine type II collagen, emulsified with an equal volume of Freund’s complete adjuvant. After 18 days, the control group received saline solution and CIA mice received type II collagen and Freund’s incomplete adjuvant. At the day 19, controls and CIA mice were divided into 2 subgroups: one receiving vehicle (n=16) and one receiving 30 mg/kg/day of tofacitinib (n=16). After 35 days the first collagen administration, the mice were sacrificed and the blood collected. [file 13075_2021_2587_MOESM1_ESM.tif]

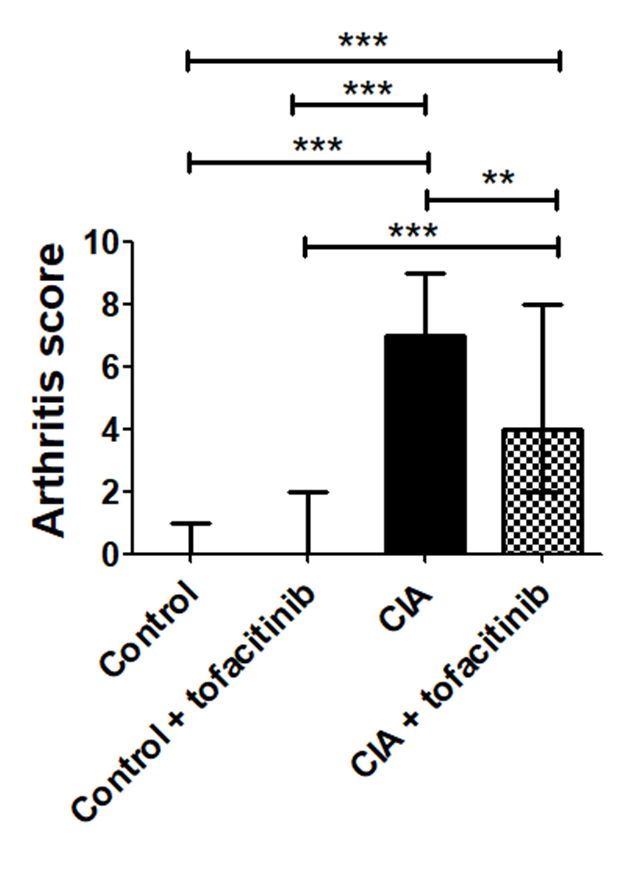

Supplement: Supplementary file 2 — Additional file 2: Supplementary material 2. Arthritis score evaluation. The histogram showed the median and the range of the arthritis score evaluated the day 35. The collagen induced a significant increase of arthritis score when compared to control group, and 30 mg/Kg/day of tofacitinib prevented the increase of arthritis score (**=p=0.001; ***= p< 0.0001). [file 13075_2021_2587_MOESM2_ESM.tif]
